# Disordered eating in anorexia nervosa: give me heat, not just food

**DOI:** 10.3389/fpubh.2024.1433470

**Published:** 2024-11-06

**Authors:** Emilio Gutierrez, Naomi García, Olaia Carrera

**Affiliations:** ^1^Department of Clinical Psychology and Psychobiology, College of Psychology, University of Santiago, Santiago de Compostela, Spain; ^2^Venres Clínicos Unit, College of Psychology, University of Santiago de Compostela, Santiago de Compostela, Spain; ^3^Hospital Unidad de Salud Mental Infanto-Juvenil, Complexo Hospitalario Universitario de Santiago (CHUS), Santiago de Compostela, Spain

**Keywords:** heat treatment, AN phenotype, activity-based anorexia, hyperactivity, leptin

## Abstract

The recommendation to apply external heat to patients with anorexia nervosa (AN) was first documented by William Gull in 1874. Gull encountered this practice during his tenure as a consultant physician, responsible for issuing medical certifications for wealthy clients seeking admission to Ticehurst Asylum, one of the most successful and reputable private asylums in England. Gull attributed the origins of this practice to the studies by Charles Chossat (1796–1875), a physiologist, physician, and politician from Geneva, who discovered the therapeutic effects of heat on starved animals by chance. In the 20th century, further evidence of the beneficial effects of heat on starved animals emerged serendipitously when anomalies were observed following a malfunction in laboratory thermostats controlling animal temperatures. Moving into the 21st century, experimental research has empirically substantiated the crucial role of ambient temperature (AT) in the animal model of activity-based anorexia (ABA). Recent translational studies have shown that a warmed environment significantly reduces anxiety around mealtime in AN patients, a method shown to be more effective than exposure-based procedures. Despite the overwhelming evidence from both animal and patient studies, it is difficult to comprehend how the impact of providing a warm environment to AN patients, particularly around mealtimes, continues to be a neglected area of research.

## Introduction

Anorexia nervosa (AN) is a mental disorder primarily characterized by a restriction of food intake and significantly low body weight, which predominantly affects adolescent women (1). Although AN has a relatively low prevalence, it leads to severe physical and psychological consequences and has the highest mortality rate among mental disorders (2). While disordered eating does not equate to a clinical diagnosis of an eating disorder, it is one of the main characteristics of AN. Disordered eating involves difficulties with the regularity, variety, flexibility, and enjoyment of normal *eating* habits, often accompanied by restrictive behaviors, preoccupations, or obsessions.

Therefore, it is a common practice in treatment programs for AN to address dietary restrictions by attempting to rapidly and fully reestablish normal eating behavior, which includes the oral intake of sufficient calories through three large meals and snacks distributed throughout the day (3). However, achieving this regular eating routine and restoring normal body weight remains a particularly challenging cornerstone of treatment. Due to the impaired ability to eat, nutrients are often provided through nasogastric tubes (4). While this is a safe method, force-feeding to accelerate weight gain offers no advantages other than the cost savings associated with shorter hospital stays (5, 6).

Furthermore, it is well known that patients with AN experience difficulty gaining weight during nutritional rehabilitation, even with a high caloric intake (7, 8). Approximately one-third of the energy consumed by these patients cannot be processed and is dissipated through elevated diet-induced thermogenesis (9, 10), making the process of weight restoration particularly challenging and uncomfortable (11).

Historically, AN patients have often justified their reluctance to eat by citing abdominal pain and discomfort, which turns the eating process into a distressing experience. This was first described by Lasegue (12): *“Feeling of immediate discomfort after eating: vague sensations of fullness, anxiety, and gastralgia, whether postprandial or already manifested from the beginning of the meal.”* p.388, translated from the original in French. A discomfort being referred to by many others later as ‘*to be troubled with indigestion*” [(13), p. 613]; “*epigastric distress*” [(14), p. 1085]; “*organic disease of the stomach (ulcer)*” [(15), p. 745]; “var*ied gastrointestinal symptoms*” [(16), p. 817]; “*indefinite gastrointestinal disturbance*” [(17), p. 681]; “*abdominal discomfort*” [(18), p. 113]; “*dull or burning pain in the epigastrium*” [(19), p. 256]; *abdominal discomfort, a common cause of complaint in the early stages of treatment*….” [(20), p. 1771]; “*constipation and minor abdominal discomfort*” [(21), p. 345], “*abdominal tenderness […] and complaints of unbearable abdominal fullness follow the ingestion of even small amounts of food*” [(22), p. 445–446], “*abdominal pain*” [(23), p. 438], and “*epigastric discomfort*” [(118), p. 594].

These abdominal discomforts often prompt patients to chronically apply localized heat to the abdominal area, which can sometimes result in the clinical manifestation of a skin condition known as *Erythema ab ignea*, meaning “redness by fire” (24–26).

## William Gull’s recommendation is to apply heat during meals

The daily self-application of heat by AN patients appears to echo William Gull’s recommendation of applying heat during meals, as presented in his seminal address on AN to the Clinical Society of London On Friday, 24 October 1873 and published the following year in the *Transactions of the Clinical Society of London* (27): ‘*I have observed that in the extreme emaciation when the pulse and respiration are slow, the temperature is below the normal standard. This fact, together with the observation made by Chossat on the effect of starvation on animals and their inability to digest food in the state of inanition without the aid of external heat, has direct clinical bearings—it being often necessary to supply external heat as well as food to patients. The best means of applying heat is to place an India rubber tube, with a diameter of 2 inches and a length of 3 or 4 feet, filled with hot water along the patient’s spine, as Dr. Newington of Ticehurst suggested* (p. 24).

As reviewed in other studies (28), Ticehurst Asylum in East Sussex was licensed as a private asylum in 1792, with Samuel Newington (1739–1811), an apothecary and surgeon, being the first representative of the Newington family in charge of the asylum, which was erected on the grounds of his house, The Vineyard. *Circa* 1870, when the asylum was run by Dr. Herbert Francis Hayes Newington (1847–1917) and his cousin, Dr. Alexander Samuel Lysaght Newington (1846–1914), William Gull acted as a consultant physician, medically certifying the wealthy clientele admitted to Ticehurst.

It must be noted that after his successful treatment of the Prince of Wales for typhoid fever, Gull was awarded the title of baronet, which elevated his social standing and granted him access to the wealthiest strata. There is evidence of William Gull visiting Ticehurst Asylum in May 1876, where he confirmed a diagnosis of general paralysis made by HFH Newington (29).

Apart from Gull’s quote, there is no information in the Ticehurst archival records (29, 30) about why this method of applying heat was adopted at the Ticehurst asylum. However, feeding patients who refused to eat voluntarily remained a constant concern.

Thus, Charles Newington (1781–1852), the second owner of the asylum, published in *The Lancet* an article titled, ‘*An instrument invented for administering food and medicine to maniacs by the mouth during a closed state of the teeth’* (31). As highlighted in his obituary in a local chronicle, Charles Newington was very ingenious. *Amongst his numerous inventions was an instrument designed to feed individuals intent on self-destruction through starvation. In its present modified form, this is still used and has never been known to fail*’ [(32), p. 55]. Continuing this tradition, Charles Newington’s grandson, Theodore Newington, published another description of an instrument developed while serving as a physician’s assistant at Bethlem Royal Hospital. The instrument, a nasal tube, allowed patients who refused to eat to be fed without *‘the necessity of having to open the mouth, which, with patients with good teeth and strong jaws, is sometimes exceedingly difficult’* [(33), p. 83]. This focus on involuntary force-feeding as a quicker method likely supplanted the use of heat and the patience required to secure the patient’s collaboration. As Silverman (34) points out, in four of eleven commentaries on the last article by Gull (119), force-feeding was advised as the optimal strategy: ‘forcible administration of nourishment so very simple a process that there need be no hesitation in resorting to it when necessary; these are at once safe and effective, and by their means nutrition can not only be carried on for an indefinite length’ [(35), p. 597]. This same confidence in forced refeeding had been expressed earlier by two doctors (Dr. Williams and Dr. Edis) during the discussion that followed Gull’s speech to the London Clinical Society on October 24, 1873, but there was no mention of the use of external heat, nor Chossat in the minutes of the meeting published in The British Medical Journal (36).

## From prizes to grants: the Montyon legacy

As Gull himself acknowledged, his advice was based on preclinical animal starvation studies performed by a physiologist, physician, and politician from Geneva, Charles Chossat, who reported as a result of his ‘animal experiences,’ as he named them in the pre-scientific language of the time, the healing effects of heat on starved animals (38).

With funds from the legacy of a prominent philanthropist, the Baron de Montyon (37), the French Academy of Sciences awarded on a second instance the 1841 Montyon Prix in experimental physiology to Charles Chossat’s main work entitled “*Recherches expérimentales sur l’inanition”* published in 1843. In his book, Chossat reports detailed observations on the consequences of starvation in different species that were readily disseminated and widely commented on in England in the first scientific dissemination book for the general public written by George H. Lewes (1817–1878): *The Physiology of Common Life* (39). In chapter VII, Volume I, entitled ‘*Why we are warm, and how we keep so’* [(122), pp. 281–315], Lewes includes a detailed description of Chossat’s starvation experiments that Lewes concluded ‘*are well known, and the results are accessible in almost every textbook’* (Smith, *ibid.*, p. 352). As Prof. Robert Boakes (40) explained, the Russian translation of *The Physiology of Common Life* made a profound impression on the adolescent Ivan Pavlov, who, as an older adults man, could still quote long sections from it. Chossat’s work advanced many of the findings now established by experimental physiology on the effects of starvation on different organs and tissues in animals starved to death. Similarly, in the two books reporting the Minnesota Starvation Experiment in the 20th century, Ancel Keys found the quantitative experimental studies of Chossat ‘*were surprisingly elaborate for the time’* [(41), p. 198]. Most notably, in Chapter 9, entitled ‘*Morphology of Some Organs and Tissues*,’ under the section ‘*The History of an Error,’* Keys and colleagues underscored the work of Chossat, referring to the erroneous assertion in physiology textbooks regarding the absence of cardiac atrophy as a result of undernutrition and starvation despite the detailed findings of Chossat (42) regarding heart atrophy in starved animals.

Unlike the plethora of strains of mice and rats that have become conventional in laboratory experimentation nowadays, Chossat’s observations included only a single rodent mammal, a guinea pig, with the rest being observations made mainly with birds (17 pigeons, 7 doves, 1 hen). In the fourth chapter of *Recherches expérimentales sur l’inanition*, entitled “*Du réchauffement artificiel*,” Chossat (42) describes in 47 pages the results of 13 *experiences* on the warming-up of 26 different animals after having been deprived of food to the very edge of death: ‘*I confess that this was not without the vivid satisfaction that I saw an animal arrived in a way by the starvation to the last term of the insensitivity, the prostration, and the cooling, to revive somehow, and to retake a big degree of force muscular and of sensitivity very quickly, and it without food, without the drink, and other help than the application of the artificial heat’,* (p. 595, translated from the original in French).

The first animal to be successfully revived was a turtle dove with 35% weight loss and a body temperature 19° C below normal. Believing the dove to be dead, Chossat disposed of it by throwing it away near the stove and was bewildered to find that ‘*The appetite comes back at the animal’s inanities that one resuscitates by the artificial warming-up; because one sees them leaving the steams and going to tickle everything that they can meet’* (1843, p. 604).

Furthermore, Chossat’s observations were even more precise, as the recovery of appetite did not necessarily imply the recovery of the animals’ digestive faculties, i.e., when the artificial reheating was suspended, the animals could not digest the food: ‘*The digestion takes place, on the contrary, while continuing the artificial warming-up during one sufficient time’* (p. 605).

Chossat not only reported the effects of rewarming on these animals but also described the active thermoregulatory behavior of the animals that sought proximity to the stove: *‘I noticed that as the animals took their strength and their temperature, that they preferred to remain perched more and more on the edge of their steams, a position that they often preserved during several consecutive hours, receiving hardly a small amount of heat. It also happened to them to leave the steams and, when they had gotten more or less cold, often one saw them bringing closer to the stove and to warm themselves against its walls’* (1843, p. 615). This active thermoregulatory behavior preceded the repeated pressing of a lever to activate a heat lamp in food-deprived rats exposed to a cold AT (43, 44). Under such experimental conditions, rats worked harder to obtain heat when deprived of food (45, 46) or when the intensity and duration of heat bursts were reduced (121). More recently, Hildebrand et al. (47) reported how rats subjected to the standard activity-based anorexia experimental setting (a 1.5-h/day feeding schedule plus free access to an activity wheel) lost weight, reduced their running activity, and spent more time resting on a hot plate heated to 37°C by a continuous flow of hot water. This preference for the hot plate prevented hypothermia. It significantly reduced wheel activity and significantly less weight loss than rats lacking the hot plate in their box. This better bodyweight conservation by animals with access to the warming plate was observed even though the cumulative food intake during the 6 days of the study did not differ from that of the animals without access to a warming plate.

In Gull’s reference to the need to add external heat during the common practice of refeeding at Ticehurst Asylum, it remains unclear whether the connection to Chossat’s work was made by a member of the Newington family at Ticehust Asylum, most of whom were medically qualified, or whether it was William Gull himself who associated Chossat’s work with the use of the heating device. Regardless of who was responsible for the connection to Chossat’s work, providing heat to patients is the first example of translating basic scientific discoveries in a laboratory into potential treatments for AN patients.

These two observations of Chossat, the facilitation of food intake by warming and the thermoregulatory behavior of starved animals, have taken a long time to be confirmed by experimental research in the animal laboratory with animals subjected to different degrees of food deprivation. Moreover, just as Chossat’s research arose from an act of serendipity, when he inadvertently threw the turtledove’s body near the stove, the malfunction of the thermostats that regulated the temperature of a laboratory was the precursor of lines of research that have provided experimental evidence for Chossat’s pioneering observations.

## Malfunctioning thermostats in the animal lab and warming ABA rats

More than a century later, this facilitating role of an unplanned warming environment was reported in an experiment assessing food intake and weight gain in male Sprague–Dawley rats subjected to enforced exercise, either treadmill or swimming (48). Twenty-four rats with an average weight of 270 g were divided into four equal groups, i.e., a control group of no swimming and three groups that differed in the amount of enforced swimming (30, 60, or 120 min in the morning and 30, 60 or 120 min in the afternoon 4 days/week for 4 weeks).

Food was provided for 18 h each day to ensure equal time availability across groups swimming for different durations. The forced swimming was highly energy-demanding, as the water level prevented the animals from resting or supporting themselves on the tank edge. This procedure led to a reduction in food intake on exercise days compared to both non-exercise days and non-swimming control rats, which is in line with the findings by Edholm et al. (49) in cadets.

On the 14th day, due to a thermostat malfunction, the AT increased from the intended 24°C to 28°–30°C for 3 days. The authors observed a puzzling effect during this period: food intake increased with the temperature rise, despite the continuation of the enforced heavy exercise schedule, with the effect being particularly significant for the 4-h swimmers.

In contrast, the facilitative effect of high AT on food intake reported by Stevenson et al. (48) appeared to be a direct one, as it did not involve decreased activity, given that constant exercise was maintained. This facilitative effect of high AT on food intake would, at first glance, seem inconsistent with Brobeck’s (50) thermostatic theory, which posits that high AT should neither increase locomotor activity nor stimulate feeding. However, while food intake in homeotherms is generally related to environmental temperature (51), this relationship does not apply to rats exposed to restricted feeding, as their hypothermic state alters this response.

Once again, serendipity highlighted the importance of AT, as demonstrated in an unpublished experiment where a change in AT occurred accidentally in the research laboratory of Kelly Lambert in the Department of Psychology at Randolph-Macon College in Ashland, Virginia (USA). Dr. Lambert was interested in the development of ulcers in animals exposed to a procedure similar to ABA, which combined restricted feeding and wheel access—referred to as “activity stress” [AS; (52)]—referred to a condition that arises when weight loss exceeds 30% of body weight (53). Owing to the malfunction of the thermostat, the laboratory temperature increased from the standard 22°C to 26.5°C, at which point the rats ceased exhibiting the typical increase in wheel activity in response to food restriction (54).

This lack of increased activity motivated a planned experiment, and the results were presented at a meeting of the Southern Society for Philosophy and Psychology in Louisville, KY, in April 1990 (55) but were not published in a scientific journal. In that unpublished experiment, 44 male, 47-day-old Long-Evans rats were weight-matched and randomly assigned to one of four groups in a 2 × 2 factorial design. The first factor referred to access to the wheel made up of two groups, Active and Sedentary, and the second factor was the environmental temperature (25°C or 19.4°C). The four groups were subjected to a restricted feeding program. While the animals in the active group only had access to food for 1 h a day, the amount of food available to the sedentary group was equal to that consumed by the active animals.

The results of that study confirmed the findings of the “accidental” study. The rats at standard temperature (19.5°C) differed significantly in activity and the number of ulcers. Furthermore, regarding survival rates, the animals in the active group at 19.5°C began to die on Day 3 of the procedure, and all had died by day 6. However, the active animals exposed to 25°C began to die on Day 5, and 3 animals survived the 14 days of the study. These differences in survival were statistically significant.

More direct experimental evidence of the beneficial effect of heat on rat survival came from another laboratory study that also focused on ulcer production. In this study, 60 Sprague–Dawley female rats were exposed to the standard AS procedure for ulcer production (restricted feeding for 1 h/day and free access to the activity wheel) after a period of acclimatization to the wheels under *ad libitum* feeding (56). AT was set at 21° ± 0.5°, and four groups of rats received a different manipulation when they reached a pre-defined criterion of imminent death. Twenty-four hours before the onset of acute hypothermia subsequent death, the animals were divided into four groups: those that continued exposure to the Activity Stress (AS) procedure (*N* = 18), those warmed with a lamp (37°C, *N* = 9), those deprived of access to the activity wheel (*N* = 13), and those that were sacrificed (*N* = 8). A fifth control group of sedentary rats (without access to the activity wheel) was maintained on the same restricted feeding schedule (1 h/day).

Sixty percent of the heat lamp group survived the experimental conditions for an additional week in this warmer environment, with a mean survival (12.2 days) even greater than that of the fifth group of controls mentioned above, who never had access to the activity wheel (11.1 days). The average survival of the heated group doubled the survival of both the animals exposed to the same experimental procedure at 21° ± 0.5° C (Group 1, 4.8 days) and the group with blocked wheels (Group 2, 5.3 days). It should be noted that both the work of Lambert (54) and Morrow et al. (56) were studies centered on AS ulcer research.

This is particularly relevant in the case of Morrow et al.’s study (1997) since the main conclusion drawn with respect to the warmed group is in relation to the formation of ulcers, and at no time does it refer to the decrease in activity or the facilitation of intake: “*Warming animals with a heat lamp 24 h prior to predicted mortality significantly increased the likelihood and length of survival and attenuated gastric erosion formation*,” (p. 823). However, Morrow et al. did not appear to be aware of the fact that the food intake of warmed rats matched that of the control non-active group, although this finding can be deduced from the data they presented in the column of Table 3 reporting food consumption on the day the rats were euthanized [(56), p. 822], which showed that food intake of warmed rats on the day rats were sacrificed did not differ significantly from that of the sedentary control group that never had access to the wheel. Moreover, although Morrow et al. do not provide it, according to data reported in Table 3, a student’s *t*-test calculated to assess between-subgroup differences on the last food intake day revealed that the food intake of heated rats was significantly higher than that of the rats with blocked wheels, *t* (20) = 2.10, *p* < 0.05. Furthermore, in Lambert and Hanrahans’ unpublished study, the food intake of rats housed at 19.5°C was lower than that of rats in the warm group. However, the difference did not reach statistical significance. However, the group housed at 25°C was well below the thermoneutral zone [27–31°C, (57)] and far away from those 37°C used in the study by Morrow et al. (56).

However, more compelling experimental evidence regarding the effect of AT on intake has been forthcoming from research on the experimental procedure known as Activity-Based Anorexia, ABA (58), a procedure similar to the above mentioned ‘Activity Stress,’ as both have their origin in the seminal work ‘Self-starvation of rats living in activity wheels on a restricted feeding schedule’ (59). Since the publication of this pioneering study, hundreds of studies have reiterated three robust outcomes: first, active animals maintained on a restricted feeding schedule and given access to a running wheel increased their physical activity excessively; second, daily food consumed by these animals was lower than the intake of animals in a sedentary control group submitted to the same restricted food schedule but without access to the activity wheel; and third, animals with wheel access showed a severe weight loss usually exceeding the standard removal criterion of 25% body weight loss in ABA studies (60).

However, these three well-established facts were reversed when the housing AT of animals exposed to ABA was increased to the thermoneutral zone [32°C; (61)]. In this study, two weight-matched groups of rats were randomly allocated to either an active standard ABA condition (1.5 h/day food access and activity wheel) or a sedentary control condition (1.5 h/day food access and no activity wheel). AT for all animals was set at 21°C. This AT remained constant for half of the rats assigned to the ABA condition until they reached the standard removal criterion of a 25% weight loss, while for the other half, the AT was raised to 32°C once they reached a 20% weight loss of baseline weight. The same AT change was performed in half of the yoked sedentary rats, which had previously matched the initial body weight of the active rats. Notably, this increase in the housing AT caused the food consumption of the active animals to be greater than that of the sedentary ones.

Furthermore, the group of active rats housed at 32°C was the only group that showed a significant increase in food intake on the final day of the study. A similar facilitating effect of AT on food was later reported in active female rats (62). Interestingly, the drop in food intake observed in sedentary male and female rats at 32°C did not result in weight loss compared to sedentary rats housed at 21°C. Moreover, male and female rats at 21°C reached the weight loss removal criterion. In contrast, the body weight of all warmed rats gradually increased, reaching the recovery criterion. This criterion was first defined in the pioneering work of Routtenberg and Kuznesof in 1967 as the body weight on any given day exceeding the weight of the animal from 4 days prior.

Similarly, a further study has shown that under limited access to food conditions, a warmer environment seemed to be more influential than food availability for body weight gain (62). In this study, rats maintained time-restricted access to food for 1.5 h/day for a week at an AT of 21°C. They were randomly allocated to a 32°AT or maintained at 21°C for two more weeks while subjected to a restricted feeding schedule. During the first week, all the animals lost weight while adapting to the restricted access to food, but during the third week, the food intake for both animals maintained at 21°C as those now housed at 32°C had reached a plateau. However, during the second and third weeks, animals maintained at 21°C consumed on average 21.5% more than animals housed at 32°C, but despite this highly significant difference in ingested food (*p* < 0.0001), only the warmed animals gained a significantly greater amount of weight. By the end of the experiment, there were no differences in body weight between the two groups despite the extra 21.5% of food consumed by the animals housed at 21°C. These results confirmed previous findings with females of a different strain, Wistar hooded rats [(63), experiment 3], where, after a loss of 20% baseline body weight, access to wheels was denied, and AT was maintained at room temperature (22°C) or increased to 29.5°C. Room temperature was insufficient to keep the rats alive, but the increase in AT to 29.5°C allowed weight recovery even though the recovering animals ingested significantly less food than those kept at 22°C. Furthermore, in this study, raising AT to 29.5°C did not depress food intake, but all warmed active animals gained weight and recovered.

In the case of the active animals, the effect of heat on food intake could be facilitated via the cancelation of the inhibitory effect of excessive activity exhibited by ABA animals, which may also be the case in the results reported by Lambert and Hanrahan, (55, 56, 61, 62). Also, in humans, the suppression of appetite induced by exercise depends on the intensity of exercise, ranging from 15 min in the case of moderate intensity to 2 h after vigorous exercise (64). However, the facilitative effect of AT on food intake can be direct, as initially reported in Chossat (42), or even independent of exercise, as shown in Stevenson et al. (48), where swimming was enforced for the animals, and in Gutierrez et al. (61, 63), where increased AT either did not depress or had a major effect on food intake and bodyweight maintenance in animals (62). In either case, the better meal efficiency and less running afforded by a warm environment seemed either to prevent rats from entering the positive feedback loop linking activity and reduced food intake or to break it once it has been established.

A plausible explanation for the beneficial effect of increased AT may lie in its buffering effect over metabolic demands for maintaining body temperature. In the case of sedentary animals, this energy saving would allow them to maintain body weight despite their reduced food intake, whereas for active animals, further saving is afforded by the fall in energy expenditure by reduced running. Notably, the increase in AT reduced the expression of Uncoupling protein one (UCP1) in the brown adipose *tissue* (BAT), and the reduction in adaptive thermogenesis, together with the reduction of hyperactivity, would account for a better preservation of body mass and even the recovery of body weight of ABA rats (65).

Is there any evidence in humans of an effect of AT similar to that just described in ABA research? The answer is clearly yes. At least five studies have described natural body weight fluctuation in patients with AN at the time of admission. In four of these studies (66–69), excepting one (70), a strong association was observed between warm and cold seasons and the body weight/BMI of AN patients, with patients having significantly lower weight on admission during the coldest months of the year compared to patients admitted to the hospital during the warmer months. It is worth noting that a recent study (69) involving a large sample of 606 patients aimed to elucidate the contradictory results of the studies of Fraga et al. (68) and Kolar et al. (70). It is striking that this systematic and significant effect of AT on the weight/BMI of patients with AN was not revealed in the literature until 2012. The explanation is very simple because, until that date, AT had been systematically ignored in the literature. However, the first reference to the probable relationship between AN and AT can be traced to an editorial in *The Lancet* on March 24, 1888, commenting on the last paper published by W. Gull on AN that appeared the preceding week in this journal, saying, ‘*Most of the cases seem to occur in the colder months of the year, and possibly this may be more than a coincidence*’ [(71), p. 584].

Likewise, until 2002, there was an absolute absence of reports in the literature on the use and abuse of saunas by patients with AN (72). This absence of reports was most striking in patients supposedly mastering the most diverse array of weight-losing strategies in their relentless pursuit of thinness. However, the use and abuse of sauna baths seems to be a practice as common as it was underreported in patients with AN (73). The discovery of the systematic neglect of these two areas of research was ascertained after experimental evidence gathered in the animal lab describing the influence of AT on ABA research, first described by Charles Chossat two centuries ago. Hence, more and more voices (74) are demanding “*to take advantage of this unique biobehavioural model [ABA] to develop and refine novel treatments for AN”* (p. 330).

Finally, there is another area where warming may help in the refeeding process central to treating AN patients. If there is any single major challenge in the treatment of AN, it is anxiety in relation to meals and weight restoration (75). Meal-related anxiety is negatively correlated to BMI (76), remains high even after weight restoration (77), and is directly correlated to outcome (78), and anxiety is one of the elements that has been proposed as a factor for the maintenance of food restriction in AN patients (77, 79).

Pre-meal anxiety levels have been associated with reduced calories ingested and reduced dietary fat consumption (77, 79). For example, in Steinglass et al.’s (77) study, the pre-meal anxiety of 23 acutely weight-restored AN patients (BMI 20.2 ± 0.8) was compared with healthy controls before undergoing three lab evaluations (one yogurt snack, one multi-item lunch, and one macaroni and cheese lunch). Pre-meal anxiety, as measured by the Spielberger State–Trait Anxiety Inventory (STAI-S), was significantly higher (*p* < 0.001) in patients as compared to healthy controls in each meal type (50.7 ± 11.8 vs. 23.3 ± 3.5, yogurt snack; 52.5 ± 12.9 vs. 25.5 ± 4.5, multi-item lunch; and 55.8 ± 10.9 vs. 25.6 ± 5.7; macaroni and cheese). In comparison with HC, pre-meal anxiety scores among AN patients were significantly correlated to intake in the multi-item macaroni and cheese lunch but not in the yogurt snack. In contrast, no relationship was observed between the amount of calories/fat ingested and post-meal anxiety (79). These findings highlight the potential usefulness of developing treatments aimed at reducing pre-meal anxiety in AN patients. One of the treatments evaluated for reducing pre-meal anxiety has been exposure and response prevention therapy (80). Thirty-two hospitalized AN patients receiving standard inpatient behaviorally based eating disorder treatment and having achieved near normal weight restoration with a BMI of >18.5 were enrolled in a randomized controlled trial comparing Exposure and Response Prevention (EXRP) with a comparative condition, Cognitive Remediation Therapy (CRT). These authors reported no differences in changes in pre-meal STAI-S between both treatments (58 + 15 at baseline to 51 ± 16 after EXRP treatment, versus 51 ± 11 to 48 ± 10 in the CRT group) but described a modest positive effect of reducing anxiety on the patients’ intake within the EXRP group but not in the CRT group. Moreover, the administration of alprazolam was ineffective in reducing anxiety before eating (81).

An alternative approach for reducing anxiety around meals consisted of music therapy (MT), as reported by Ceccato et al. (82). In this study, 24 patients (age 17.4 ± 3.3 and BMI 15.7 ± 2) attending an Eating Disorders Unit in Italy voluntarily participated over a period of 6 months (19 sessions in total) in a program of inpatient treatment that included weekly 1-h group TM sessions led by a qualified music therapist every Wednesday before dinner was served. Throughout those 6 months, on Mondays, Tuesdays, and Wednesdays, anxiety was measured before dinner using an anxiety thermometer, a Visual Analogue Scale that goes from 0 to 100, previously validated against a measure of generalized anxiety [GAD-7, (83)], with a sample of 228 patients with mixed cardiovascular conditions (84). MT’s activities were both active and receptive. The authors reported that after participating in the MT group, anxiety before meals was significantly lower on Wednesdays at 52.1 ± 6.4 compared to Mondays at 57.7 ± 6.2 or Tuesdays at 55.6 ± 6.4.

A different method with an immediate effect on reducing anxiety in AN patients has been described by Zandian et al. (85). In this study, immediately after lunch, 18 AN patients (mean age, 16.5 years and mean BMI, 14.3) rested individually for half an hour in bed in a small 6 m^2^ room either heated to 32°C, or at 21°C or together with other patients in a shared room (70 m^2^) also at 21°C. All patients underwent these three conditions in random order and at intervals of 2 days. Patients filled out the STAI-S four times: 1.5 h before and immediately before the break, immediately after the break, and 1 h after. Pre-meal STAI-S anxiety scores 1.5 h before meals were always approximately 55–60, but patients resting individually at 32°C experienced a significant reduction in their STAI-S scores (approximately 35) at both times of post-meal measurements while in the communal condition. There were no changes. For those patients resting individually at 21°C, there was a transient reduction during the first 0.5 h of rest time, but anxiety almost returned to baseline levels at the last measurement point of 1.5 h after resting. These results coincide with the relaxing effect associated with the use of thermal vests (3 h/day) observed in another clinical trial carried out in 2004 (86). In this study, the patients reported experiencing a calming effect, in addition to an improvement in digestion associated with the use of heat, which was no small thing considering that gastrointestinal discomfort is very common among AN patients, as well as being the most common postprandial distress syndrome (87) and one of the causes of calorie restriction (77, 88).

Once again, a review of the literature can explain the process by which supplying heat around meals is a useful strategy for managing anxiety during the refeeding process due to the elevated cortisol levels associated with anxiety in AN (89, 115). Elevated serum cortisol levels in AN were first reported in 1966 (90), and several studies have reported high cortisol levels in AN patients (91, 92), with both fasting and postprandial cortisol levels being negatively associated to subjective measures of appetitive drive (89). Fraga et al. (93) have shown that increasing housing AT after 20% body weight loss not only reversed the hyperactivity and weight loss pattern characteristic in animals exposed to ABA but also reduced the corticosterone level (ng/mL) of heated rats to a fifth of the level found in ABA animals at a standard AT of 21°C (1466.87 ± 125.28 and 273.05 ± 43.77, respectively, for animals housed at 21°C or 32°C). Thus, future randomized clinical trial studies should explore whether providing heat to AN patients at mealtimes (both before and after meals) can neutralize the effects of sustained HPA axis hyperactivity in AN and facilitate the nutritional rehabilitation of AN patients.

## Concluding remarks and future research directions

At this point, it is necessary to clarify what type of disorder we have in mind throughout this paper. William Gull’s (27) description of the AN disorder includes three characteristics: hyperactivity, severe weight loss, and restricted feeding. From an evolutionary perspective, this AN phenotype may be considered a conserved response to starvation (94, 95).

This evolutionary phenotype fits well with the present DSM-5 criteria for AN diagnosis (1). Besides meeting criterion A for weight loss, the AN phenotype fulfills both criterion B *(“persistent behavior that interferes with weight gain, even though the patient’s weight is already significantly low”’)* and criterion C (“…or *persistent lack of recognition of the seriousness of the current low body weight”*). Thus, together with criterion A, both B and C criteria recognize the behavioral activation and lack of concern in the AN phenotype (96, 97), where hyperactivity functions as an alternative thermoregulatory strategy with respect to the more common response to famine consisting of passive “shallow torpor,” which includes reduced activity, resulting in energy conservation (98).

From this perspective, both the requirements of “*intense fear of gaining weight or becoming fat*” (Criterion B) and “*disturbance in the way in which one’s body weight or shape is experienced, undue influence of body weight or shape on self-evaluation*” (Criterion C), which conform current mainstream thinking since DSM-III (117), could be considered superfluous in retaining the essence of the AN phenotype (28, 99, 114). This highlights the relevance of Gerald Russell’s remarks (116) concerning the modern phenomenology of AN: “*The dread of fatness is likely to be a modern development in the psychopathology of anorexia nervosa. It need not persist in future generations of anorexic patients. The time may be approaching when it will be advisable to retreat from our cherished diagnostic criteria of anorexia nervosa, as there may be a false precision in the current formulation*” (p. 10). Hence, the new epidemy of atypical AN diagnosis does not need to meet the A criterion of DSM-5 but rests exclusively upon these modern conceptualizations of the psychopathology of anorexia nervosa as reflected by criterion B, “*Intense fear of gaining weight or becoming fat*,” and criterion C, “*Disturbance in the way in which one’s body weight or shape is experienced, undue influence of body weight or shape on self-evaluation*.”

Thus, to rely excessively on self-declared patient motivations around weight/fatphobia and body image disturbances to justify the positively valued weight loss process is, at best, a recent cultural coloring of the AN entrapment/entanglement (100–102), which does not exclude the role of the active acculturation process spread through the therapeutic milieu and the media (99). Furthermore, the developmentally low leptin levels in young females have been considered fundamental for the AN psychological and behavioral manifestations (hyperactivity, anxiety obsessiveness, and so on) that characterize this state of entrapment/entanglement. Moreover, although there are mixed preclinical data on the efficacy of leptin treatment in *reduced semi-starvation-induced hyperactivity* in semi-starved animals (93, 103), recent employment of recombinant leptin in AN patients seems promising (104–106).

Nevertheless, the maintenance of the psychopathological profile of AN in terms of weight/fatphobia and body image disturbances in the diagnostic criteria from the DSM-III to the present DSM-5, presumably grasping the essence of the disorder, has not been accompanied by progress in the development of an effective treatment, either psychological or pharmacological, despite the increasing sophistication of treatments (28, 107, 108).

In this paper, we propose an alternative conception of the disorder and an alternative approach to treatment based on the review of strong preclinical evidence. Furthermore, in AN patients, hyperactivity is inversely related to AT (67); the probability of menses recovery during the warmer months of the year is twice as high as in autumn or winter, even though patients’ body weight was 2 kg less in the warm season than in the cold season (109); sauna use by AN patients has been underreported in the last 150 years (73) and no adverse effects of sauna use by AN patients has been reported in the literature (72); and finally the worldwide stable distribution of references to anorexia nervosa over the last 25 years reported in two bibliometric studies (references to AN in the literature were associated to higher but not extreme latitudes, i.e., 40–55° latitude range in the Northern Hemisphere) in climates with regular seasons and no severe temperature variations across seasons (110, 111). Collectively, this preclinical and clinical evidence enables certain clear predictions on the effect of supplying heat with respect to the three main characteristics of the disorder: restrained eating, weight loss, and hyperactivity. In other words, the predictions regarding what should and should not happen if AN patients are supplied external heat during treatment are as follows:

1. Hyperactivity should decline and not increase with increased AT up to the thermoneutral zone.
2. Eating would be facilitated at a lower cost (suffering) for the patient. Unlike normal-weight people who decrease food intake when AT increases, AN patients would increase their ingestion.
3. Weight gain will be enhanced with increased AT up to the thermoneutral zone. In terms of body weight gain during nutritional rehabilitation, increased AT would be more crucial than an increase in calories *per se*. By keeping the intake of calories constant, in comparison with a colder environment, a warmer environment would result in greater weight gain.
4. In addition, anxiety, depression, irritability, and sleep quality and quantity would also improve when AN patients are supplied with external heat.

These predictions about what should and should not happen if AN patients are supplied heat during treatment can be easily attested (112). Though hundreds of studies have assessed the putative efficacy of an array of pharmacological treatments without a shred of evidence supporting them, there has been a conspicuous lack of studies examining the effects of heat supply in AN treatment, i.e., a case series (120), two randomized clinical trials studies (86, 113), and a study on the anxiolytic effect of a warmed room (85). Thus, the majority of the aforementioned predictions are either unattested or partially tested.

The systematic use of warmed rooms is one of the four cornerstones of treatment at Mandometer Clinics^1^, and undoubtedly, these clinics are best positioned to perform future research following either a dismantling strategy to determine the efficacy of heat on the outcome of the full treatment package or a dose–response research strategy manipulating exposure times to warmed rooms. These are just a few examples that would allow us to advance our understanding of the role of heat supply in the treatment of AN patients, as first recommended by William Gull.

## Coda

In 2013, the Marqués de Valdecilla Hospital in northern Spain moved the eating disorders unit to a new, more modern building where each room had an independent temperature control. By chance, I was visiting the new location of the eating disorders unit when the unit director, who was familiar with our research on the beneficial effects of heat, told me a curious anecdote of what happened the first day the patients were transferred to the new facilities. They spontaneously selected a 32°C temperature setting on the thermostat in the communal room where they spent most of the day. When he took me to see the room, we were surprised to find that the nursing staff had placed a note covering the thermostat warning against any tampering in the selection of the room temperature. At present, 11 years later, a note still stands under the thermostat warning patients against any unauthorized manipulation of the thermostat.

This anecdote illustrates how, inexplicably, the impact of providing a warm environment to AN patients, particularly around mealtimes, continues to be a neglected area of research.

## Footnotes

^1^https://mando.se/en/mandometer-method/the-four-cornerstones/
